# Topographic Distribution of Stimulus-Specific Adaptation across Auditory Cortical Fields in the Anesthetized Rat

**DOI:** 10.1371/journal.pbio.1002397

**Published:** 2016-03-07

**Authors:** Javier Nieto-Diego, Manuel S. Malmierca

**Affiliations:** 1 Auditory Neuroscience Laboratory, Institute of Neuroscience of Castilla y León (INCYL), Salamanca, Spain; 2 Salamanca Institute for Biomedical Research (IBSAL), Salamanca, Spain; 3 Department of Cell Biology and Pathology, Faculty of Medicine, University of Salamanca, Salamanca, Spain; McGill University, CANADA

## Abstract

Stimulus-specific adaptation (SSA) in single neurons of the auditory cortex was suggested to be a potential neural correlate of the mismatch negativity (MMN), a widely studied component of the auditory event-related potentials (ERP) that is elicited by changes in the auditory environment. However, several aspects on this SSA/MMN relation remain unresolved. SSA occurs in the primary auditory cortex (A1), but detailed studies on SSA beyond A1 are lacking. To study the topographic organization of SSA, we mapped the whole rat auditory cortex with multiunit activity recordings, using an oddball paradigm. We demonstrate that SSA occurs outside A1 and differs between primary and nonprimary cortical fields. In particular, SSA is much stronger and develops faster in the nonprimary than in the primary fields, paralleling the organization of subcortical SSA. Importantly, strong SSA is present in the nonprimary auditory cortex within the latency range of the MMN in the rat and correlates with an MMN-like difference wave in the simultaneously recorded local field potentials (LFP). We present new and strong evidence linking SSA at the cellular level to the MMN, a central tool in cognitive and clinical neuroscience.

## Abstract 2

This study of higher-order auditory cortex strengthens the case for long-latency stimulus-specific adaptation as a genuine neural correlate of the mismatch negativity, which flags salient stimuli.

## Author Summary

Sensory systems automatically detect salient events in a monotonous ambient background. In humans, this change detection process is indexed by the mismatch negativity (MMN), a mid-late component of the auditory-evoked potentials that has become a central tool in cognitive and clinical neuroscience over the last 40 years. However, the neuronal correlate of MMN remains controversial. Stimulus-specific adaptation (SSA) is a special type of adaptation recorded at the neuronal level in the auditory pathway. Attenuating the response only to repetitive, background stimuli is a very efficient mechanism to enhance the saliency of any upcoming deviant or novel stimulus. Thus, SSA was originally proposed as a neural correlate of the MMN, but previous studies in the auditory cortex reported SSA only at very early latencies (circa 20–30 ms) and only within the primary auditory cortex (A1), whereas MMN analogs in the rat occur later, between 50 and 100 ms after change onset, and are generated mainly within nonprimary fields. Here, we report very strong SSA in nonprimary fields within the latency range of the MMN in the rat, providing empirical evidence of the missing link between single neuron response studies in animal models and the human MMN.

## Introduction

A critical function of the brain is to identify uncommon and potentially important stimuli while ignoring irrelevant ambient backgrounds [1–3]. In humans, this ability is reflected by an electrophysiological brain response called mismatch negativity (MMN), a mid-late (150–200 ms) deflection of the auditory event-related potentials (ERP) that is elicited by uncommon, but not by repetitive, sounds [4–7] and serves to automatically redirect attention toward potentially relevant stimuli [8]. Importantly, the MMN signal is altered in patients with schizophrenia and other psychiatric disorders and can be used as an index of cognitive decline in normal and pathological neurodegenerative processes [9,10]. The MMN has been extensively studied using the “oddball” paradigm, in which infrequently occurring sounds, i.e., “deviant” tones, are randomly interspersed among frequent monotonous sounds, i.e., “standard” tones. MMN studies have advanced our knowledge on many aspects of change and novelty detection, but scalp recordings limit our ability to pinpoint its regions of generation.

Recent studies over the past decade have taken advantage of the oddball paradigm to study adaptation in single auditory neurons. Stimulus-specific adaptation (SSA) may be a counterpart phenomenon to MMN that is studied in single neurons using this paradigm [11]. As in MMN, neurons showing SSA adapt to frequently occurring stimuli (standards) yet respond strongly to rare stimuli (deviants). Within the auditory system, SSA was originally reported in the primary auditory cortex (A1) [12] as a higher level of adaptation to a specific stimulus, different from firing rate adaptation resulting from changes in the intrinsic properties of the neuron. SSA shares many properties with the MMN, and it is important because it may be a neural correlate of the MMN, or at least one of its early generators [11,13]. The basic properties of SSA have been studied in great detail not only in A1 but also in the subcortical inferior colliculus (IC) [14–16] and medial geniculate body (MGB) [17,18]. One important difference between SSA in the auditory cortex and subcortical stations is their anatomical location. SSA is strong and widespread only in the nonlemniscal regions of the IC and MGB [16], while SSA has been described as strong and widespread in lemniscal A1 [12,19]. However, detailed studies on SSA within the different cortical fields beyond A1 are lacking. Since SSA is stronger in the nonlemniscal regions of the IC and MGB, it is reasonable to hypothesize that SSA in the nonprimary regions of the auditory cortex would also be stronger than in A1. Indeed, previous studies on the general response properties of the auditory cortex reported that nonprimary neurons in the cat [20,21] and rat [22–24] auditory cortex adapt more strongly than in A1. Even studies in human subjects have shown differential adaptation between primary and nonprimary cortical areas [25–27]. Moreover, two recent studies that mapped auditory ERPs in the rat showed robust MMN-like responses in nonprimary auditory cortical fields [28,29].

The main goal of the present study was to generate a complete and fine-grained map of SSA across all known cortical fields in the rat. Despite interspecies differences, the rat auditory cortex shares many common anatomical and physiological features with other species [23,30,31], including primary and nonprimary regions. Primary regions of the auditory cortex are characterized by a thick, dense, granular layer and receive major layer IIIb/IV thalamocortical projection from the first-order (or lemniscal) auditory thalamus. The nonprimary auditory cortex is formed by surrounding regions that subsequently process input from primary regions and receive major layer IIIb/IV projection from the higher-order (or nonlemniscal) auditory thalamus [31]. Detailed electrophysiological mapping studies [23,24,32] have identified at least five tonotopically organized fields in the rat auditory cortex. The A1, the anterior auditory field (AAF), and the ventral auditory field (VAF) are all considered primary fields [23,33]. Additionally, two distinct nonprimary regions have been identified: the posterior auditory field (PAF), located in the dorsocaudal border of A1; and the suprarhinal auditory field (SRAF), in the ventral margin of the auditory cortex [23,31,34,35]. Unfortunately, there are no specific stains or molecular markers that cause one cortical region in the rat to stand out unambiguously from another, but they show a robust organization of multiple response properties that follow a particular spatial organization [23,36]. Our results demonstrate that, although SSA is indeed present in A1 and the other two primary fields, it is markedly stronger in the nonprimary fields PAF and SRAF, consistent with the SSA observed in nonlemniscal parts of the IC and MGB. Another important finding in our data is that SSA observed in auditory cortex is robust up to 200 ms after stimulus onset, well within the latency range of the MMN-like potentials in the rat [37]. These data suggest the existence of a hierarchically organized system for SSA processing [13] and reinforce the notion that nonprimary SSA is a more direct neural correlate of the MMN than the SSA observed in A1.

## Results

**Fig 1:**
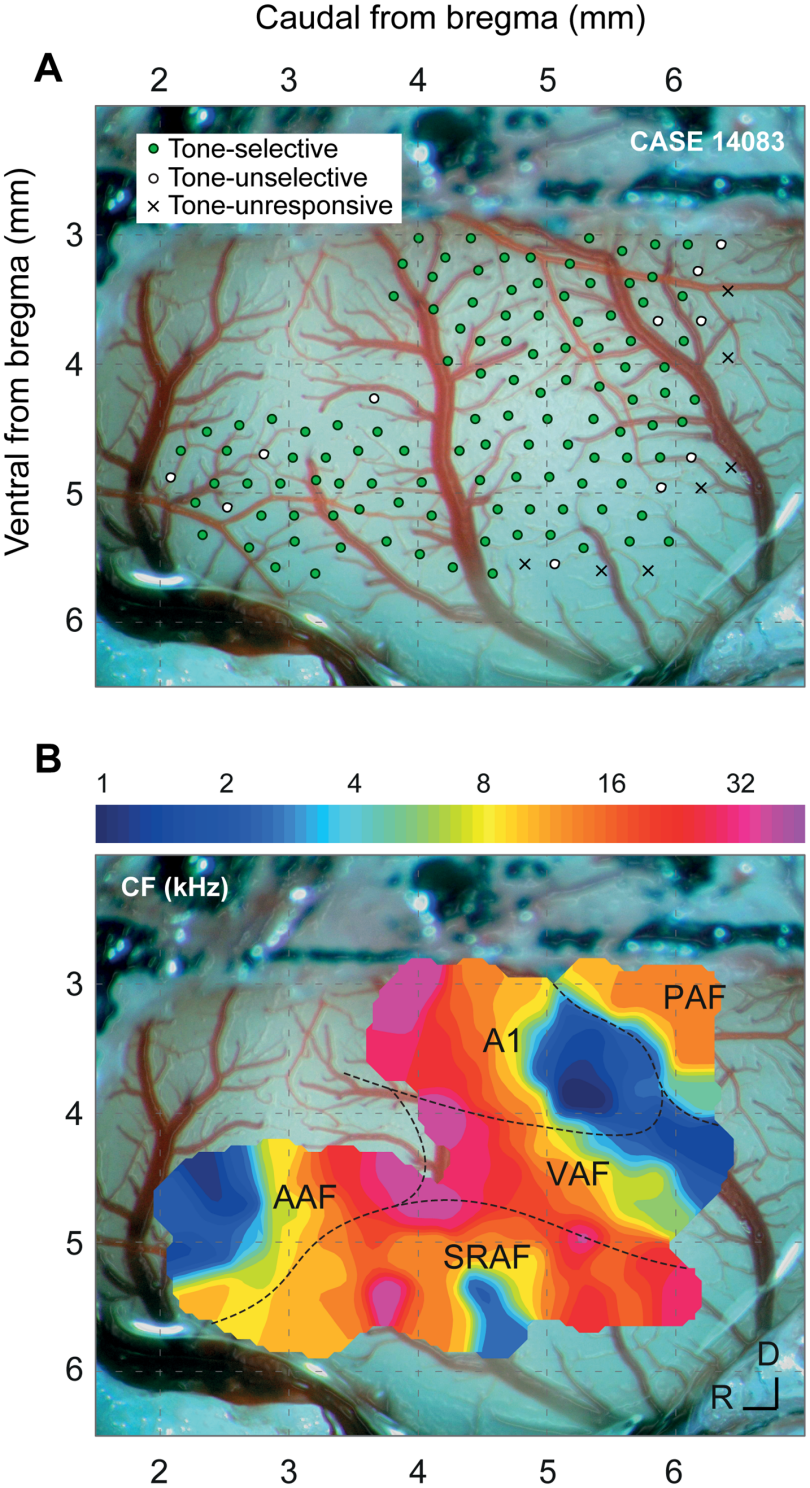
Experimental setup. **A.** Sample case with 132 MUA recording sites from layers IIIb/IV throughout the cortical fields in one representative animal. At every site, the CF was determined (if possible), and we presented an oddball paradigm (c.f., Fig 2). Sites are classified according to pure-tone selectivity (Selective: tone-responsive with a clear CF; Unselective: tone-responsive, but with a lack of a clear CF; Unresponsive: no significant responses to pure tones). **B.** Outline of the different cortical fields in this particular case, as derived from the tonotopic gradients. Each field shows a characteristic CF gradient [23], with A1 being the most easily identifiable.

To study the topographic distribution of SSA across the auditory cortex, we recorded a total of 816 multiunit activity (MUA) clusters from layers IIIb/IV within all cortical fields from the left auditory cortex in 12 animals (total number of recordings by field: A1, 167; AAF, 121; VAF, 164; SRAF, 169; PAF, 119). Local field potentials (LFPs) were simultaneously recorded from the same electrode in four of the animals. In each animal, we made a microelectrode mapping (15–25 tracks/mm^2^) covering at least three fields (Fig 1A shows an example with 132 recording sites from all fields). Most recordings (89%) were made between 300 and 600 μm in depth, corresponding to cortical layers IIIb/IV [31]. Five auditory cortical fields were identified according to tone frequency response topographies. The limits and relative position of the auditory fields were determined for each animal at the end of the experiment, using the characteristic frequency (CF) gradient as the main reference landmark (Fig 1B). We consistently observed distinct tonotopic gradients within the different fields [23,32,36], with a high-frequency reversal between VAF and AAF (rostrally), a low-frequency reversal between A1 and PAF (dorsocaudally), and a high-frequency reversal between VAF and SRAF (ventrally). We identified the boundary between A1 and VAF as a 90°-shift in the CF gradient in the ventral low-frequency border of A1, and the boundary between A1 and AAF as an absence of tone-evoked responses in the ventral, high-frequency border of A1 (Fig 1B). We used these boundaries to assign each recording to a given field.

**Fig 2:**
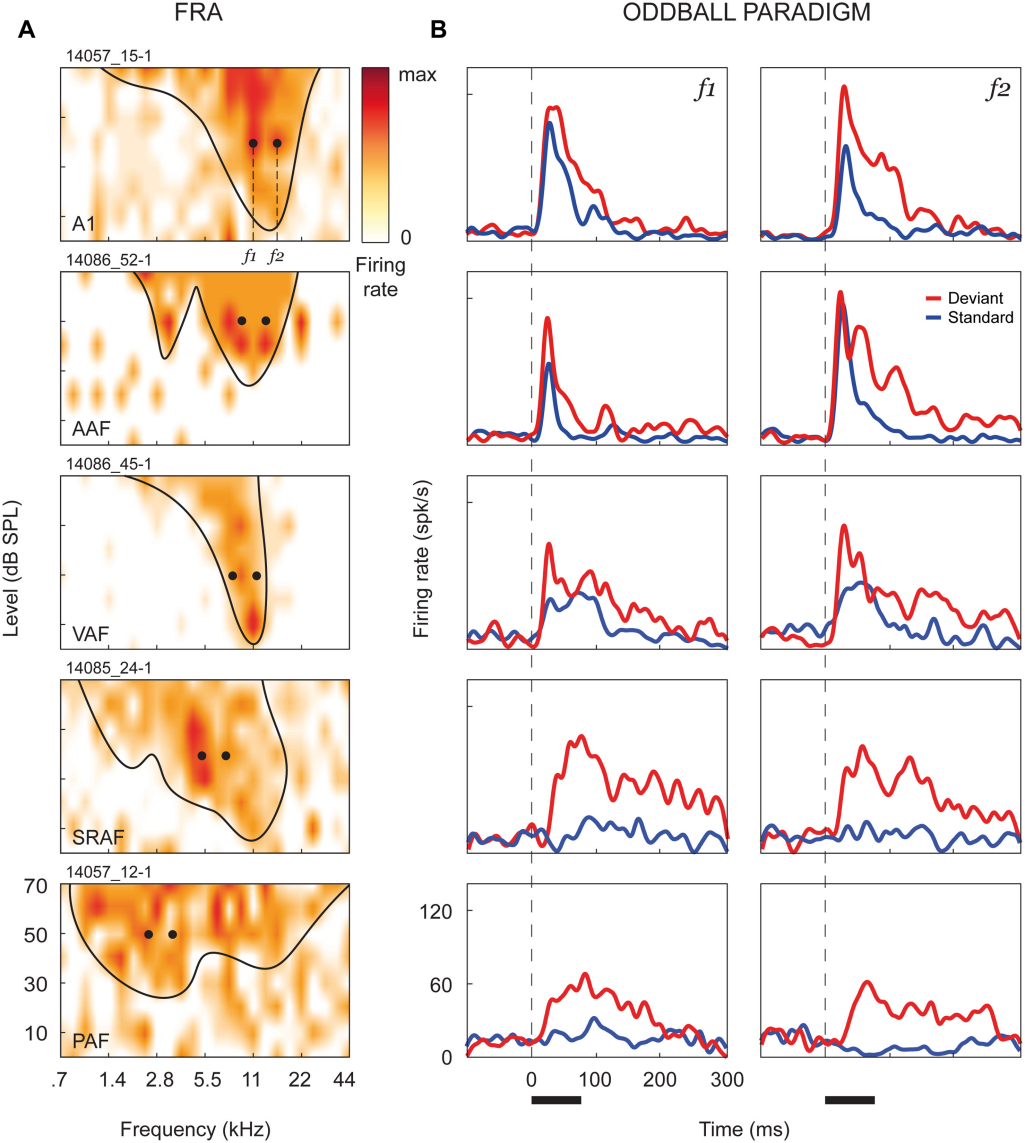
Stimulation paradigm. **A.** Representative FRAs from each auditory cortex field. Firing rate (red shading, normalized to max response) is represented as a function of frequency and intensity of the tones presented, and the frequency-tuning curve has been outlined (minimum sound intensity that elicits a firing rate over 20%–40% of the maximum firing for each frequency, excluding isolated “islands” of spontaneous activity). We selected a pair of frequencies, separated by 0.5 octaves, that elicited responses of similar magnitude at 20–30 dB above threshold. These frequencies were then presented within an oddball paradigm (250 tones, 10% deviants, 300 ms onset-to-onset interval, 75 ms tone duration). **B.** Corresponding responses to the oddball paradigm. Each plot compares spike-density functions (see Materials and Methods) in response to the same frequency, computed from the 25 deviant trials (red) and the 25 standard trials just preceding a deviant (blue). Responses to standard tones were significantly reduced in all fields as compared to deviants, but this adaptation is much stronger in the nonprimary fields (SRAF and PAF). Black horizontal bar: stimulus duration.

At every recording site, the frequency response area (FRA) was computed, and we presented an oddball paradigm (two sequences of 250 trials, 10% deviants, 300 ms onset-to-onset interval, 0.5 octaves frequency separation) using a pair of pure tones from the FRA, at 20–30 dB above CF threshold, which elicited clear responses of similar magnitude. Fig 2 shows representative MUA recordings from each auditory cortex field. Fig 2A shows their FRAs and the pair of stimuli *f*_*1*_ and *f*_*2*_ selected for the oddball paradigm, and Fig 2B shows comparative responses to each frequency when presented as either standard (blue) or deviant (red) in the oddball paradigm.

### SSA Is Stronger in Nonprimary Fields

**Fig 3:**
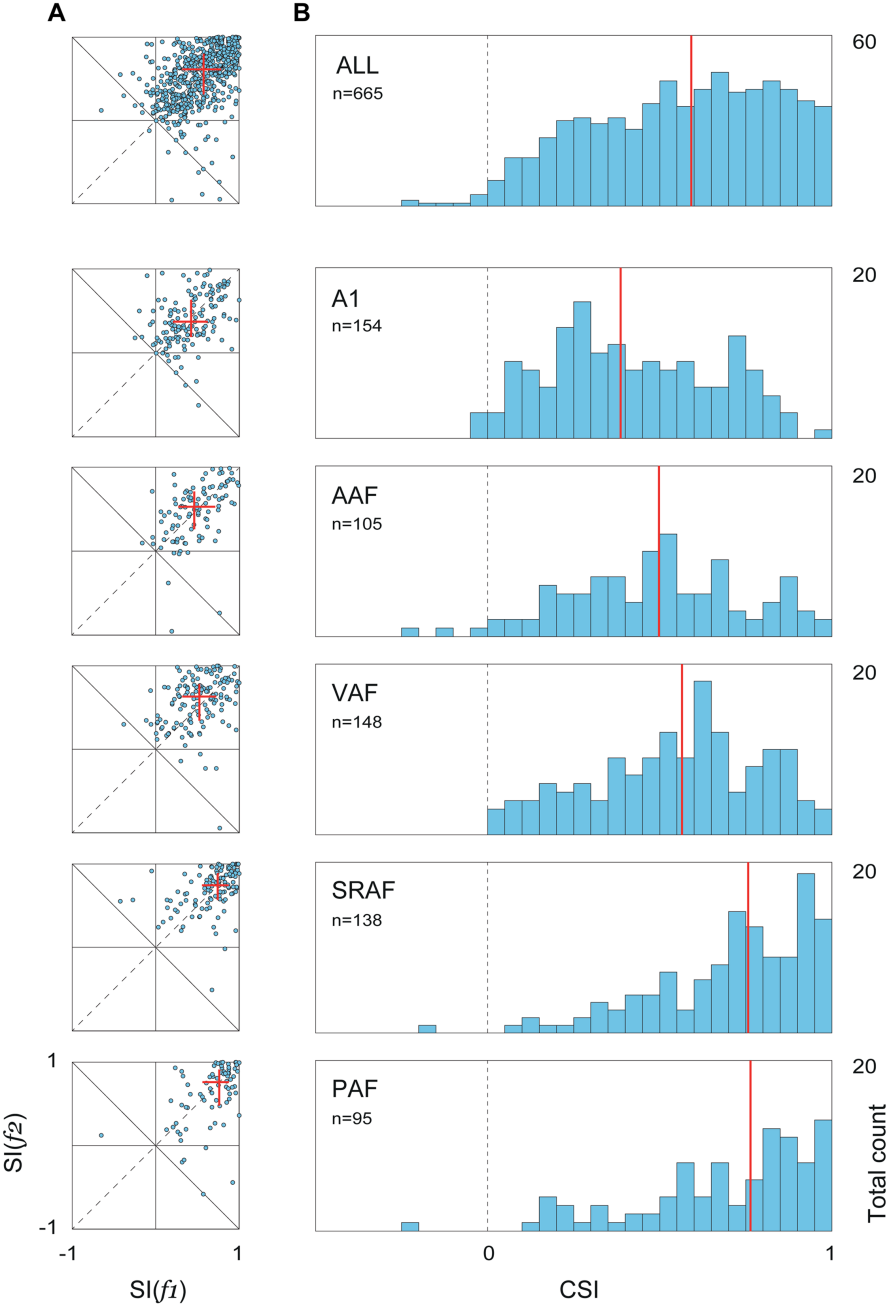
Distribution of SSA indexes within each field. **A.** Distribution of frequency-specific SSA indexes for the whole population and for each field separately. Red lines represent median and inter-quartile range for SI(*f*_*1*_) and SI(*f*_*2*_), showing a progressive increase in SSA from primary to nonprimary fields. **B.** Corresponding distributions of the CSI. In the primary fields, distributions are symmetrical and centered in medium-CSI values, but in the nonprimary fields, CSI distributions are sharply skewed to extreme levels of SSA. Red lines show distribution medians, which were statistically different between every primary and nonprimary field (see text). Underlying data for this figure can be found in S2 Data.

The main aim of this study was to quantify and compare SSA levels between the five cortical fields. Thus, we computed the stimulus-specific adaptation index (SI) for each stimulus, SI(*f*_*1*_) and SI(*f*_*2*_), and the common SSA index (CSI) for every recording site, using baseline-corrected spike counts during stimulus presentation (5 to 80 ms from stimulus onset; see Materials and Methods). Fig 3A shows a series of scatterplots illustrating the joint distribution of SI(*f*_*1*_) and SI(*f*_*2*_), for the whole population and for each field separately, and Fig 3B illustrates corresponding histograms of CSI distributions (total number of recording sites included in this analysis, as detailed in Materials and Methods, are also indicated). In all cases, points are symmetrically clustered around the main diagonal, with no significant differences between the median SI(*f*_*1*_) and SI(*f*_*2*_) for any field (paired Wilcoxon signed rank test, *p* > 0.1 in all fields), indicating that adaptation was equal on average for *f*_*1*_ and *f*_*2*_. The drift of the population medians toward the upper-right corner (Fig 3A) reveals a gradual shift of the cloud of points, from A1 to PAF fields, toward higher levels of SSA. The global population shows a CSI distribution that is slightly skewed to the right (Fig 3B, top panel). The origin of this skewness emerges once we split these distributions into the five cortical fields: the CSI distributions for the primary fields, especially A1 and AAF, are more symmetrical, centered on medium CSI values, and span the full range of possible values (Fig 3B). The same distributions for the nonprimary fields, SRAF and PAF, on the other hand, are clearly asymmetric, sharply skewed to the right toward the extreme positive CSI values, with a virtual absence of low CSI values. Moreover, the center of the distribution progressively moves to the right (i.e., toward higher CSI values) from A1 to PAF, (CSI, [Q_1_, median, Q_3_]: A1, [0.22, 0.38, 0.61]; AAF, [0.32, 0.50, 0.68]; VAF, [0.39, 0.56, 0.72]; SRAF, [0.57, 0.76, 0.90]; PAF, [0.56, 0.76, 0.89]), with the median CSI in every primary field being significantly smaller than in every nonprimary field (Kruskall-Wallis test, χ^2^(4) = 121.43, *p* < 5×10^−24^).

Correcting for baseline activity was required to measure the actual evoked response, given the high spontaneous rates seen in many recordings, particularly from the nonprimary fields (spontaneous firing rate, mean ± SEM: A1, 8.2 ± 0.7 spk/s; AAF, 7.3 ± 0.6 spk/s; VAF, 10.7 ± 0.7 spk/s; SRAF, 9.2 ± 0.6 spk/s; PAF, 13.0 ± 1.0 spk/s). This correction may have a major impact when using a contrast index such as the CSI [38], so that higher CSI values in nonprimary fields could result in part from this procedure. Therefore, we repeated the CSI calculation using the absolute spike counts for the same time window. As expected, all CSI values were overall reduced, but the same trend was observed between fields, since median CSI in all fields were lower than in SRAF; only CSI levels in PAF were differentially affected, so that they were no longer higher than in primary fields (CSI without baseline correction, [Q_1_, median, Q_3_]: A1, [0.14, 0.24, 0.40]; AAF, [0.18, 0.30, 0.45]; VAF, [0.21, 0.32, 0.42]; SRAF, [0.26, 0.39, 0.52]; PAF, [0.17, 0.25, 0.42]). However, given the higher spontaneous rate relative to evoked activity seen in PAF, uncorrected CSI does not faithfully represent the strong SSA (i.e., contrast) clearly observed in responses from this field (Fig 2B). Therefore, we kept using these corrected measures for the rest of the analyses.

Consistent with previous studies [23], nonprimary fields showed longer response onset latencies than primary fields for both deviant (mean ± SEM: A1, 11.6 ± 1.2 ms; AAF, 11.1 ± 1.1 ms; VAF, 17.3 ± 1.5 ms; SRAF, 27.0 ± 2.0 ms; PAF, 23.9 ± 2.2 ms; Kruskal-Wallis test, χ^2^(4) = 152.78, *p* < 10^−31^) and standard tones (A1, 16.7 ± 1.7 ms; AAF, 22.5 ± 3.3 ms; VAF, 29.8 ± 3.5 ms; SRAF, 45.8 ± 4.7 ms; PAF, 50.0 ± 8.0 ms; χ^2^(4) = 77.59, *p* < 10^−15^). From these figures, it is apparent that onset latency was significantly delayed for standards as compared to deviants in all five fields (onset latency difference, standard–deviant, mean ± SEM: A1, 7.6 ± 1.5 ms; AAF, 13.6 ± 3.1 ms; VAF, 18.0 ± 3.1 ms; SRAF, 23.2 ± 3.8 ms; PAF, 31.8 ± 7.2 ms; all significantly greater than zero, Wilcoxon signed rank test, *p* < 0.01 in all cases). Thus, in addition to an overall reduction in spike counts, SSA also produced a delay in onset latency to the standard tones. Furthermore, this delay was significantly longer in nonprimary fields than in primary fields A1 and AAF (Kruskal-Wallis test, χ^2^(4) = 34.13, *p* < 10^−6^).

### SSA Is Topographically Organized in the Auditory Cortex

**Fig 4:**
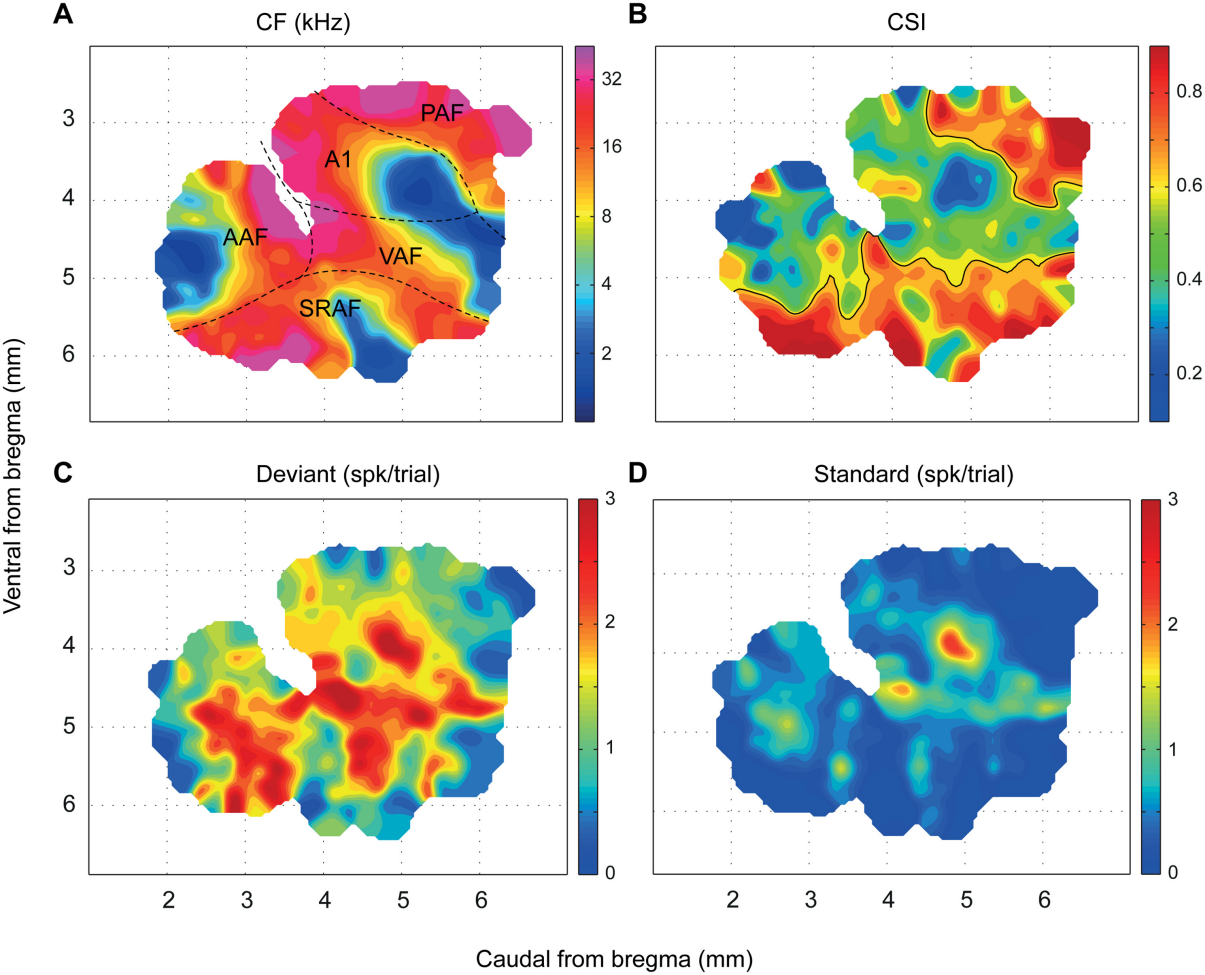
Topographic distribution of SSA throughout the auditory cortex. **A.** Synthetic map of the auditory cortex showing the location of the five cortical fields. The CF was used as the main reference to put into register the individual maps from the 12 animals. The high topographical correlation of the CF (see text) confirmed the robustness of the alignment. **B.** Topographic distribution of SSA in the auditory cortex. The CSI follows a statistically significant topography within the auditory cortex (see text), with the highest values being confined to the nonprimary fields. **C,D.** Topographic distribution of the responses to deviant and standard tones, respectively, from which the CSI was computed. Responses to standard tones were almost zero in the nonprimary fields. Data underlying these maps can be found in S3 Data.

The sharp differences in SSA levels observed between primary and nonprimary fields derive from a distinct topographic organization of adaptation throughout the whole auditory cortex (Fig 4). The absolute position of the map with respect to bregma differed between animals by up to 0.6 mm, but the relative position and orientation of the five cortical fields were highly conserved from one animal to the next. Thus, we constructed a synthetic map of CSI from all available data. Using the CF gradient as the main reference landmark, an appropriate shift was applied to each map to maximize the degree of CF coincidence between them (Fig 4A; cf. Fig 1 in [23] and Fig 1 in [36]). We quantified the quality of the alignment as the local coincidence of CF values. The resulting correlation of CF between neighboring sites was next to maximal (Topological product, *P*_*T*_ = 0.9686, permutation test, *p* < 0.001) [39]. Fig 4B shows the CSI map, while Fig 4C and 4D show the corresponding maps of the response to deviant and standard stimuli (within the stimulus-fitted window), from which the CSI was computed. The CSI follows a statistically significant topographic distribution (Topological product, *P*_*T*_ = 0.2342, permutation test, *p* < 0.001), meaning that neighboring sites are likely to have more similar CSI values than more distant ones. To better determine the nature of this topography, we traced a boundary following the median iso-CSI contour (Fig 4B; median population CSI = 0.60) whenever this line enclosed a region of area greater than 0.5 mm^2^. This procedure revealed an emergent organization of SSA, showing a large region of low-to-medium CSI values that covers the central and rostral portions of the auditory cortex and two separate and distinct high-CSI regions confined to the posterodorsal and ventral margins of the map, respectively (Fig 4B). Remarkably, the CSI-based boundary that defines the posterodorsal high-CSI region matches almost perfectly the boundary between A1 and PAF previously traced from the CF gradient reversal (Fig 4A). Similarly, the iso-CSI contour that separates the ventral high-CSI region matches very well the caudal SRAF/VAF and rostral SRAF/AAF boundaries.

Finally, these high-SSA regions revealed in Fig 4B can be seen also as regions of extremely low spike count to the standard stimuli in Fig 4D. Indeed, the “CSI” and “Standard” maps are almost complementary, such that regions of extreme CSI values correspond to those with virtually no response to standard stimuli, while regions of low-medium CSI match those with significant response to standards. This observation reveals a strong CSI dependence on the standard response being low, rather than on the deviant response being high. In fact, CSI was negatively correlated with both deviant (DEV) and standard (STD) response strength, yet much more strongly to the standard (Spearman correlation coefficient, ρ[CSI,DEV] = −0.19, *p* < 10^−6^; ρ[CSI,STD] = −0.81, *p* < 10^−152^). This also indicates that CSI values tend to be higher for neurons with an overall lower firing rate, as confirmed by a subsequent analysis (*v*.*i*.).

### SSA Occurs at the Late Component of the Response

**Fig 5:**
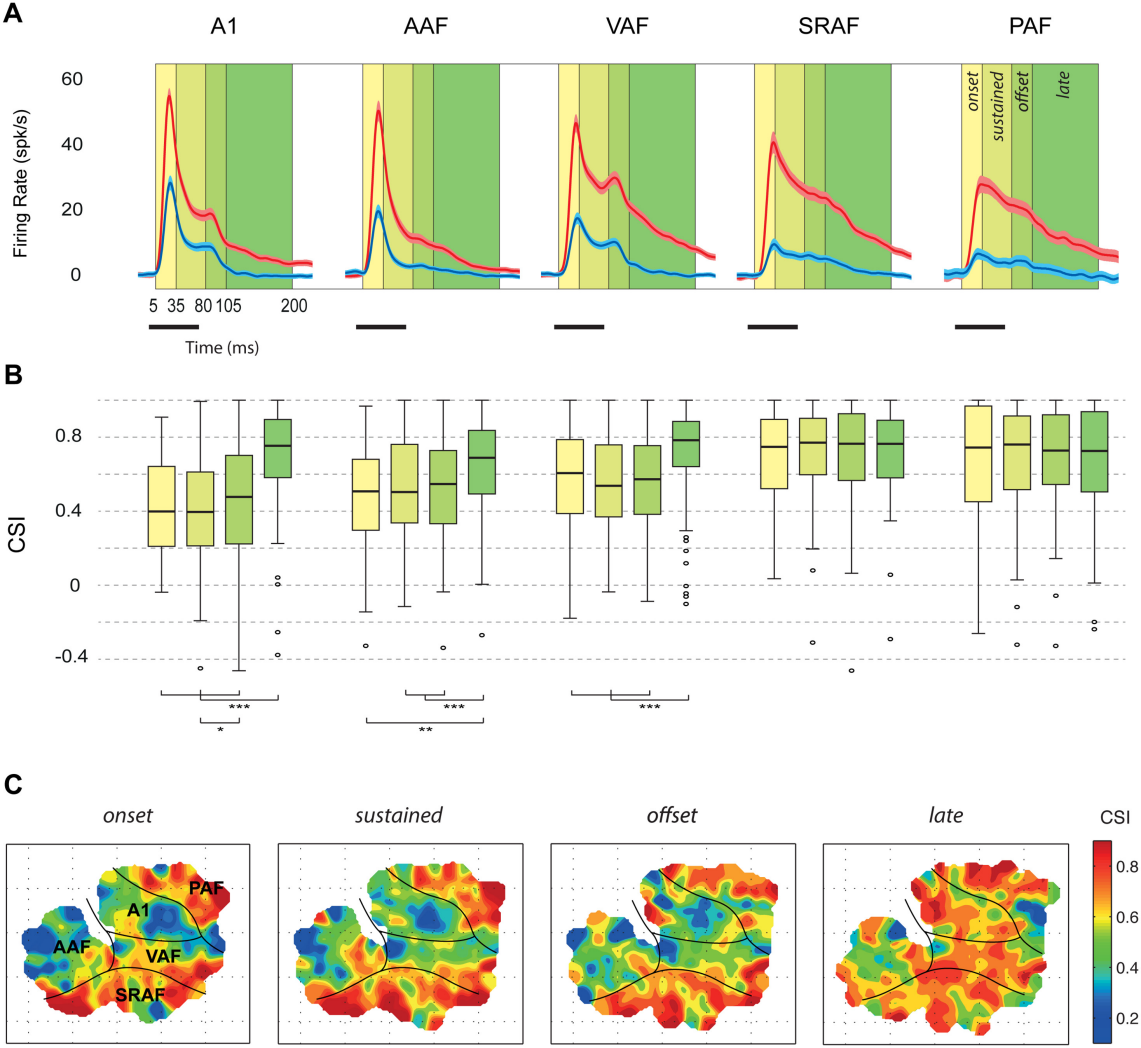
Variation of the CSI throughout the neural response. **A.** Grand-average responses (baseline-corrected firing rate, mean ± SEM) to standard (blue) and deviant (red) tones within each field. Many recordings showed significant responses beyond 100 ms from stimulus onset. **B.** Distribution of CSI values (thick bar: median, box: interquartile range, whiskers: full range excluding outliers) computed at different time windows with respect to stimulus presentation. In the nonprimary fields, SSA was high through the entire response. In primary fields, median CSI was lower than in nonprimary fields from onset to offset components but not for the late component, which showed CSI levels as high as in the nonprimary fields. **C.** Topographic distribution of SSA for the four different time windows. Note that only the late-component CSI is high throughout the entire auditory cortex. Underlying data for this figure can be found in S4 Data.

SSA was suggested as a potential neural correlate for the MMN, but previous studies neglected an analysis of the responses to deviant and standard tones at different temporal courses during stimulus presentation and beyond. Since we observed responses of long durations to deviant tones in many recordings (deviant response offset, mean ± SEM: A1, 162.6 ± 5.7 ms; AAF, 149.8 ± 6.9 ms; VAF, 194.2 ± 4.6 ms; SRAF, 196.4 ± 4.4 ms; PAF, 167.9 ± 7.4 ms), we wanted to further investigate the variation of the CSI across different components of the neural response. Hence, we computed baseline-corrected spike counts for different time intervals after stimulus onset (Fig 5A): onset (5–30 ms), sustained (30–80 ms), offset (80–105 ms), and late (105–200 ms). Corresponding CSI distributions and their topography for these different time windows are shown in Fig 5B and 5C, respectively.

First, we compared median CSI between fields for every time window separately. For the onset, sustained, and offset components, we found the same trend already observed for the stimulus-fitted response window: the median CSI in every primary field was significantly lower than in every nonprimary field, and lowest of all in A1 (Fig 5B; Kruskall-Wallis test, onset: χ^2^(4) = 73.95, *p* < 10^−14^, sustained: χ^2^(4) = 109.81, *p* < 10^−22^; offset: χ^2^(4) = 60.95, *p* < 10^−11^). The CSI for the late component of the response, however, behaved differently. At this time window, there were no significant differences in SSA between fields (Fig 5B; Kruskall-Wallis test, χ^2^(4) = 7.78, *p* > 0.1).

Then, we compared CSI levels within each field for the four time windows to analyze the trend of SSA throughout the different response components. Within nonprimary fields, we found no significant differences between median CSIs measured at the four different time windows (Fig 5B; Friedman test, SRAF: χ^2^(3) = 5.03, *p* > 0.1; PAF: χ^2^(3) = 4.72, *p* > 0.1). By contrast, a highly significant window effect was found for the three primary fields (Friedman test, A1: χ^2^(3) = 109.58, *p* < 10^−22^; AAF: χ^2^(3) = 18.18, *p* < 0.001; VAF: χ^2^(3) = 55.3, *p* < 10^−11^). Post-hoc comparisons revealed that this effect was due to a specific increase of CSI at the late component (Fig 5B), with no significant differences between median CSI measured at the onset, sustained, or offset components of the response, except for a slightly significant increase from the sustained to the offset component in A1, consistent with the overall trend. Therefore, SSA in the nonprimary fields is maintained high throughout the entire response (Fig 5B and 5C). By contrast, SSA in the primary fields is moderate during stimulus presentation, followed by a specific enhancement in late components (Fig 5B and 5C), in which SSA reaches the same levels found in nonprimary fields.

### SSA Depends on Neuronal Firing Rate and Frequency of Stimulation

**Fig 6:**
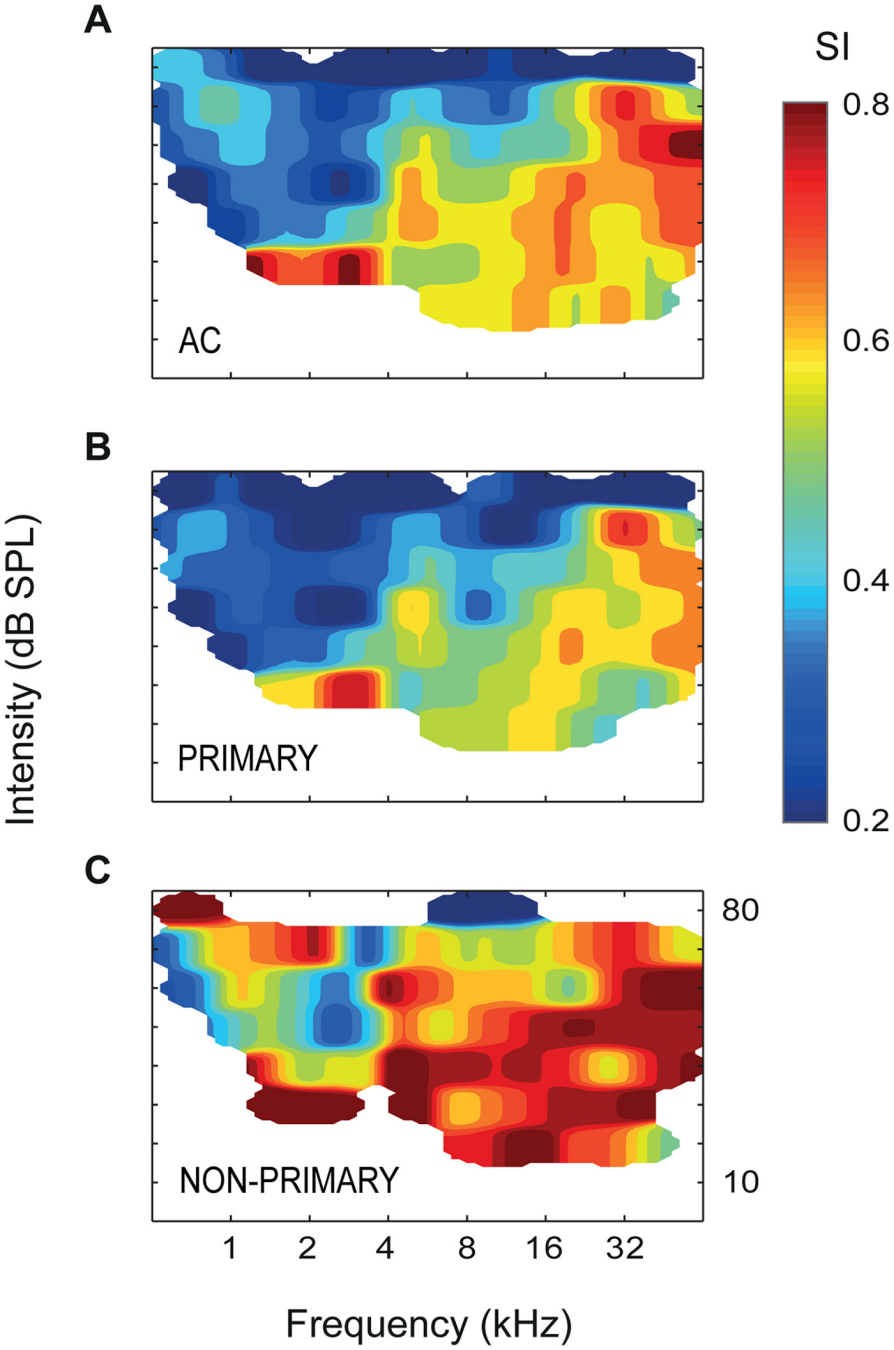
Dependence of SSA on the frequency and intensity of stimulation. **A.** Averaged SI for different values of frequency and intensity used in the oddball paradigm, for the whole set of recordings throughout the auditory cortex. SSA is significantly higher for high frequencies and low intensities of stimulation. **B.** The same effect of frequency and intensity on SSA is apparent when using all data from primary fields alone, with a virtual absence of SSA for low frequencies and high intensities of stimulation. **C.** In the nonprimary fields, this frequency and intensity dependence is weaker than in the primary fields. Underlying data for this figure can be found in S5 Data.

Upon visual inspection, regions with lowest SSA in the CSI landscape seemed to coincide with low-CF regions of the auditory cortex, particularly within A1 (Fig 4A and 4B). Since a strong dependence of SSA on frequency and intensity of pure-tone stimulation has been shown in the IC [15], we wanted to test whether a similar dependence was present in the auditory cortex. Fig 6A shows a spotlight-average map of the SI across all frequency/intensity combinations tested in the whole set of recordings. High SSA is sharply skewed toward the high frequencies and low intensities of stimulation. When we analyzed primary and nonprimary fields separately (Fig 6B and 6C), we observed that this dependence of the SI on frequency and intensity was more evident within primary (Fig 6B) than nonprimary fields (Fig 6C). Additionally, average firing rate had a topographical distribution in the dataset and was different between cortical areas (Fig 4C and 4D). Since firing rate may also have a strong impact on the amount of adaptation [17], the topography of SSA could result in part from a topography of firing rates. Finally, the observed effect of stimulus intensity on the SI (Fig 6) might be an indirect consequence of the effect of firing rate, with higher intensities of stimulation producing higher firing rates and, therefore, lower SSA.

To address these observations quantitatively, we fit a multivariate linear regression model for the SI, following a stepwise strategy (“fitlm” function in Matlab, with robust fitting options; sample data used to fit this model can be found in S5 Data). First, we used average spike count (*SPK*, as the sum of average response to deviant and standard stimuli) and frequency of stimulation (*OCT*, in octaves with respect to 1 kHz) as predictors. The resulting model was:

$$\text{SI}=\;0.51-0.046\cdot SPK+0.057\cdot OCT\;\left({\text{F}}_{2,1215}\;=\;166,p\;<\;5\times 10^{-64}\right).$$

This model accounted for 21.3% of the variability of the SI, but, more importantly, it provided a specific quantification of each effect: on average, SI decreases 0.046 points per spike of the response, while it increases 0.057 points per octave of the stimulus. Then, we added intensity of stimulation (*SPL*, in dB SPL) to the model, obtaining:

$$\text{SI}=0.72-0.051\cdot SPK+0.050\cdot OCT-0.003\cdot SPL\;\left({\text{F}}_{3,1214}=122,p<5\times 10^{-69}\right).$$

Thus, SI is also negatively correlated to intensity of stimulation. This model, however, explained 23% of the variability of the SI, only 1.7% more than the previous one. Therefore, most of the dependence of the SI on *SPL* is already explained by its dependence on *SPK*, confirming the fact that higher intensities produce lower SSA because of a higher firing rate. Therefore, we removed *SPL* from the model and replaced it with *FIELD* as a categorical factor. Now, the explanatory power of the model increased to 30.6%, mainly due to overall higher SI in the nonprimary fields:

$$\text{SI}=0.41+0.12\cdot VAF+0.24\cdot SRAF+0.20\cdot PAF-0.04\cdot SPK+0.05\cdot OCT\;\left({\text{F}}_{6,1211}=90.6,p<10^{-94}\right).$$

According to this model, mean SI is 0.41 in A1 and AAF (not significantly different from each other), 0.53 (0.41 + 0.12) in VAF (*p* < 5×10^−9^), 0.65 in SRAF (*p* < 5×10^−28^), and 0.61 in PAF (*p* < 5×10^−16^), and this difference cannot be explained by differences in firing rate within fields, since the *FIELD* factor explains an extra 9.3% of the SI variability. Note also that these are mean values and, therefore, lower than the median values shown in Fig 3, given the rightward skewness of the distributions.

As a final step, we tested this model for interactions between *FIELD* and the other three predictors separately, and we found significant interactions only between *FIELD* and *OCT*:

$$\text{SI}=0.19-0.24\cdot VAF+0.36\cdot SRAF+0.36\cdot PAF+0.078\cdot OCT-0.042\cdot VAF\cdot OCT-0.031\cdot SRAF\cdot OCT-0.034\cdot PAF\cdot OCT\;\left({\text{F}}_{9,1208}=43.8,p<5\times 10^{-68}\right),$$

indicating that the effect of frequency was weaker in VAF (*p* < 0.005), SRAF (*p* < 0.05), and PAF (*p* < 0.05) than in A1 and AAF. Therefore, the dependence of SSA on firing rate (and, indirectly, on intensity of stimulation) is comparable among the five fields, but the observed dependence of SSA on frequency of stimulation is mainly due to the fact that A1 and AAF show lower SSA for low frequencies of stimulation, as illustrated in Figs 4A, 4B and 6B. Incidentally, A1 and AAF are the cortical fields that show the most clear tonotopic gradient, each the mirror reversal of the other (Fig 4A) [23].

Since frequency and intensity of oddball stimulation were selected according to the frequency tuning and threshold of each recording site, and since there is a tendency for tuning bandwidth in auditory cortex to decrease as a function of CF [40,41], differences in SSA between fields could simply reflect differences in tuning bandwidth or CF threshold in the auditory cortex. To check this possibility, we analyzed the correlation between CSI and frequency tuning characteristics in our sample. Distributions of tuning bandwidth and threshold in our sample were consistent with previous mapping work in the rat [23]. Particularly, PAF and AAF featured the broadest tuning bandwidth and highest response thresholds (bandwidth 30 dB above threshold, in octaves, mean ± SEM: A1, 1.89 ± 0.06; AAF, 2.30 ± 0.1; VAF, 1.75 ± 0.06; SRAF, 1.98 ± 0.08; PAF, 2.95 ± 0.16; CF threshold in dB SPL, mean ± SEM: A1, 23.7 ± 0.9; AAF, 29.3 ± 1.3; VAF, 14.8 ± 0.9; SRAF, 22.5 ± 1.1; PAF, 28.3 ± 1.3). Both bandwidth and threshold in AAF and PAF were different from the other fields, but not from each other (Kruskal-Wallis test, bandwidth: χ^2^(4) = 55.60, *p* < 5×10^−11^; threshold: χ^2^(4) = 96.03, *p* < 10^−20^). By contrast, CSI was 50% higher in PAF than in AAF, as already shown (Fig 3B). Similarly, CF threshold in VAF was significantly lower than in A1 or AAF, but the median CSI was not different between these primary fields (Fig 3B). Indeed, correlation between CSI and either tuning bandwidth or threshold was extremely weak in our sample (Spearman correlation coefficient: ρ[CSI,BW30] = 0.083, *p* = 0.04; ρ[CSI,THR] = −0.09, *p* = 0.02). These considerations demonstrate that the distinct topography of SSA that we have found is genuine and not an artifactual effect of differences in other response properties between cortical fields.

### Different Time Course of Adaptation in Primary and Nonprimary Fields

**Fig 7:**
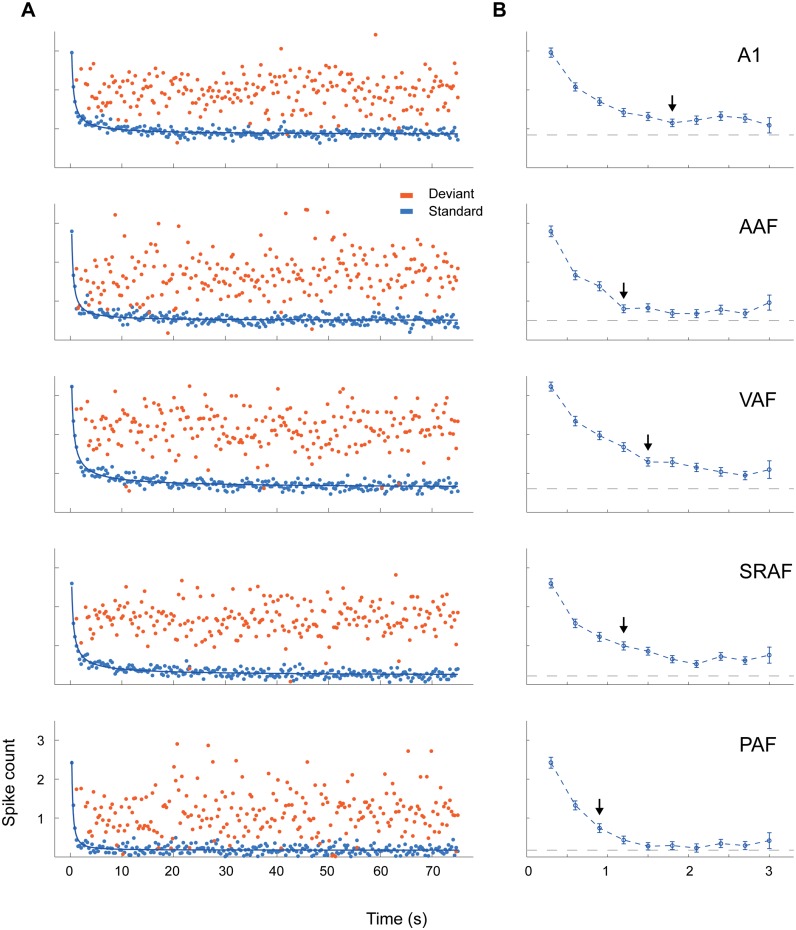
Time course of adaptation within each field. **A.** Average responses within each auditory cortex field in relation to the order of tone presentation, plotted for standard (blue) and deviant (red) tones separately. The course of standard responses over time followed a power law (thick blue lines), indicating that SSA matches stimulus statistics at many timescales. **B.** Detail of the average (mean ± SEM) standard responses for the first 10 presentations within the sequence. The arrows indicate the trial number in which the response has fallen significantly below half of the response to the first tone presentation. Gray dashed lines indicate the steady-state plateau reached by standard responses at the end of the sequence (constant parameter of the power-law fit). Adaptation occurred faster in PAF than in any other field (see text), and reached a much lower plateau in nonprimary than in primary fields. Underlying data for this figure can be found in S6 Data.

In order to study the dynamics of adaptation to the repetitive stimuli over time, we averaged responses to standard and deviant stimuli across recordings for every trial number within the sequence and plotted them in relation to the time elapsed since the beginning of the sequence, separately for each field (Fig 7A). Then, we fitted these responses to different simple models. None of the models tested could explain any amount of the variance of the deviant responses, indicating that deviant responses did not show dependence on trial number within any field. In sharp contrast, a power law model of three parameters, *y(t) = a · t* ^*b*^ *+ c*, yielded very good quality fits for the responses to standards in all fields, explaining about 80% of their variability (adjusted r^2^: A1, 0.80; AAF, 0.74; VAF, 0.84; SRAF, 0.83; PAF, 0.69) and indicating that SSA in all fields matches stimulus statistics at many timescales [42].

The most obvious difference between fields was that nonprimary fields reached a much lower plateau at their final steady-state responses (gray dashed line in Fig 7B; *c* parameter (spk/trial): A1, 0.84; AAF, 0.50; VAF, 0.60; SRAF, 0.22; PAF, 0.17; all significantly different from each other as derived from the 95% confidence intervals reported by the “fit” function in Matlab). Also, according to this model, adaptation was fastest in PAF, slowest in VAF, and not significantly different between the other three fields (*b* parameter: A1, –0.78; AAF, –0.93; VAF, –0.68; SRAF, –0.73; PAF, –1.32). This result indicates a distinct high sensitivity of PAF to repetitive stimuli, needing only a few presentations to reach its fully adapted state. This phenomenon can be readily appreciated when analyzing the responses to the first 10 standard trials of the sequence (Fig 7B). Responses to standards in the nonprimary fields adapt below half their initial strength with three (PAF) or four (SRAF) presentations of a stimulus (black arrows in Fig 7B), whereas in the primary fields it takes up to six (A1) presentations to produce this same relative reduction. Therefore, adaptation occurs faster and is stronger in nonprimary than in primary fields.

### SSA in the Auditory Cortex Correlates with the Difference Wave of the Local Field Potentials

**Fig 8:**
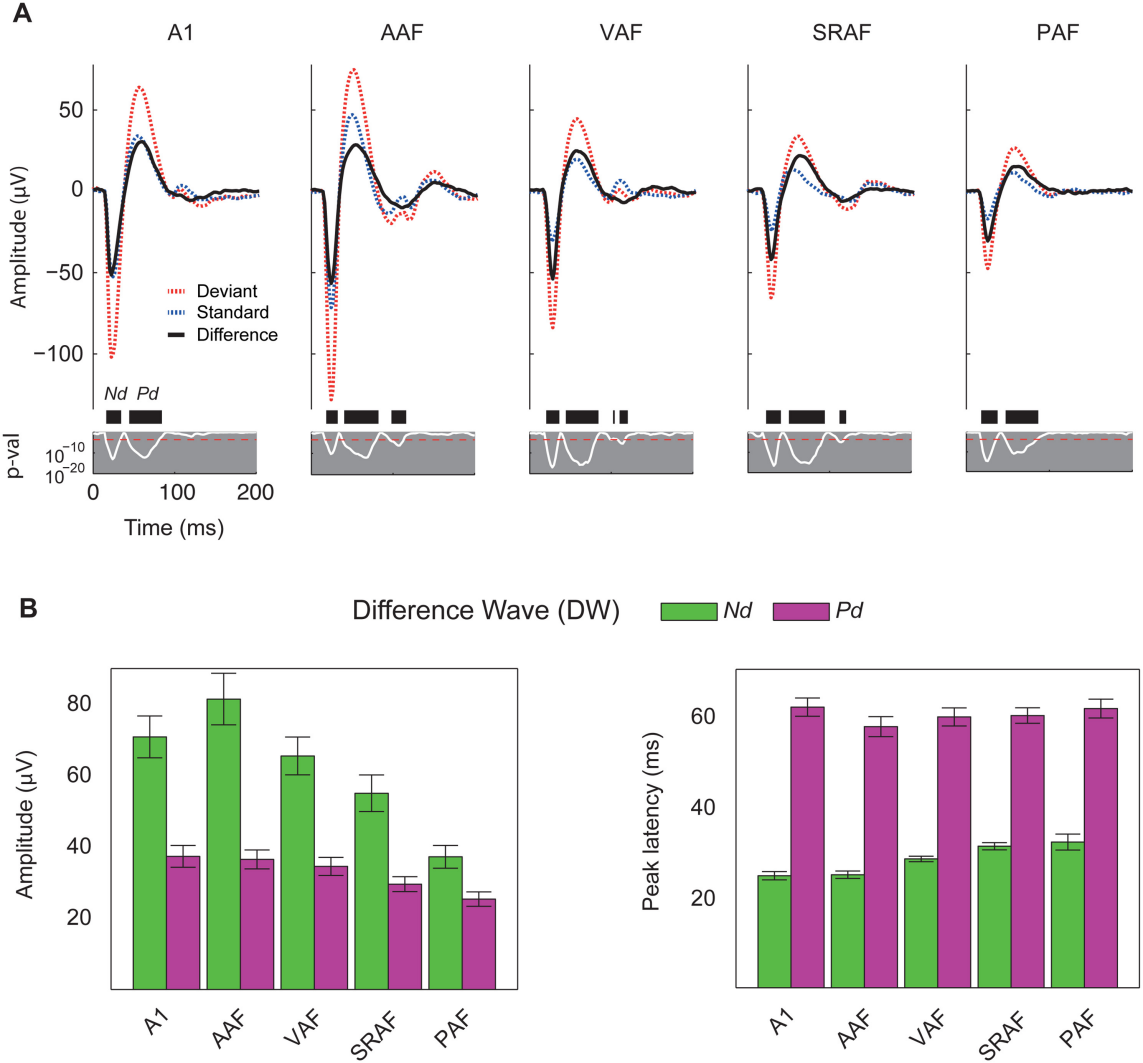
Adaptation in the LFP. **A.** Grand-average LFP traces in response to deviant (red) and standard (blue) tones, and the resulting difference wave (black), averaged for each cortical field separately. Two components of the difference wave (DW) were analyzed: the fast negative deflection (Nd) and the slower positive deflection (Pd). Note also a small but significant deflection of the LFP at longer latencies (>100 ms) in anteroventral fields (AAF, VAF, and SRAF). White line: *p*-value of the DW. Black thick bars: time intervals showing a significant DW. Red dotted horizontal line: Bonferroni-corrected critical *p*-value (bilateral *t* test). **B.** Peak amplitude and latency (mean ± SEM) of the Nd and Pd components of the DW within each cortical field. Note that the mean of the amplitudes/latencies of the individual DW components are not equal to the peak amplitude/latency of the same component in the grand-averaged DW. Underlying data for the charts in panel B can be found in S7 Data.

Whereas SSA in spike responses is a local measure at the neuron level, the MMN is a large-scale brain potential. One reasonable way to bridge this gap is to probe the correlation between adaptation of neural responses and LFP, which represent average synaptic activity in local cortical circuits [43]. Thus, we recorded LFP simultaneously with MUA in four out of the 12 animals, with a total yield of 268 recording sites (A1, 49; AAF, 48; VAF, 55; SRAF, 54; PAF, 42; Unlocalized, 20). We averaged the recorded LFP waveforms evoked by standard and deviant tones for each field separately and computed the difference wave (DW) at every time point after stimulus onset (Fig 8A). In all five cortical fields, these potentials showed the typical morphology in response to pure tones [44,45], with a fast negative deflection (Nd) followed by a slower positive deflection (Pd). These two components were present in responses to both standard and deviant tones, but their amplitudes were, in all cases, smaller for the standards, giving rise to a DW of similar shape but varying amplitudes (Fig 8A). For each recording, the peak amplitude and peak latency of the DW was measured for the Nd and Pd components, within a time window in which the DW reached statistical significance at the whole population level (16–37.6 ms for Nd and 41.5–86.7 ms for Pd, respectively, paired *t* test, Bonferroni correction for 268 comparisons, *p* < 0.05).

Peak amplitude of the DW at the Nd component showed a clear trend to be larger in primary than in nonprimary fields, being significantly smaller in PAF than in the three primary fields and smaller in SRAF than in AAF (Fig 8B; one-way ANOVA, F_4,243_ = 8.24, *p* < 5×10^−6^). This trend was still present, albeit much less clear, for the Pd component of the DW, being significantly smaller in PAF than in A1 and AAF but not different between the other fields (Fig 8B; one-way ANOVA, F_4,243_ = 3.74, *p* < 0.01). Thus, the fast Nd component of the DW showed a topographical distribution within the auditory cortex, whereas the slower Pd component of the DW showed a more homogenous distribution across cortical fields. A similar pattern was apparent for the peak latencies of each of these components (Fig 8B). The Nd component of the DW peaked earlier in the primary than in the nonprimary fields, significantly so between A1 or AAF and SRAF or PAF (mean ± SEM: A1: 24.6 ± 0.9 ms; AAF: 24.8 ± 0.8 ms; VAF: 28.3 ± 0.6 ms; SRAF: 31.1 ± 0.8 ms; PAF: 32.0 ± 1.7 ms; one-way ANOVA, F_4,243_ = 11.78, *p* < 5×10^−8^). Peak latencies for the Pd component, on the other hand, were not statistically different between fields (mean ± SEM: A1: 61.7 ± 2.0 ms; AAF: 57.4 ± 2.2 ms;, VAF: 59.5 ± 2.0 ms;, SRAF: 59.8 ± 1.7 ms; PAF: 61.4 ± 2.1 ms; one-way ANOVA, F_4,243_ = 0.70, *p* = 0.59). The steady progression of the Nd peak latency is consistent with a bottom-up propagation of the signal from primary to nonprimary fields, whereas the homogeneity of the Pd peak latency suggests a stronger contribution of intracortical processing and reciprocal interaction between fields.

To facilitate a more direct comparison between SSA for the MUA and for the LFP components, we also computed CSI values for the Nd and Pd peaks of the LFP (S8 Data). Overall, SSA at both components of the LFP was appreciably lower than for the MUA (paired signed rank test for the whole set of recordings with LFP; CSI-Nd versus CSI-onset, z-score = 6.98, *p* < 5×10^−12^; CSI-Pd versus CSI-sustained, z-score = 10.12, *p* < 5×10^−24^), but it followed the same trend to be lower in the primary than in nonprimary fields (Median CSI-Nd: A1, 0.32; AAF, 0.31; VAF, 0.45; SRAF, 0.50; PAF, 0.47; Kruskall-Wallis test, χ^2^(4) = 21.12, *p* < 5×10^−4^. Median CSI-Pd: A1, 0.25; AAF, 0.24; VAF, 0.33; SRAF, 0.37; PAF, 0.40; Kruskall-Wallis test, χ^2^(4) = 13.09, *p* < 0.05). Furthermore, CSI-Nd and CSI-Pd were strongly correlated with their corresponding CSI values at comparable time windows (Spearman correlation coefficient: ρ[CSI-Nd, CSI-onset] = 0.66, *p* < 10^−40^; ρ[CSI-Pd, CSI-sustained] = 0.43, *p* < 5×10^−12^; ρ[CSI-Pd, CSI-offset] = 0.21, *p* < 0.005).

## Discussion

In this account, we compared the level of SSA in primary and higher-order auditory cortex to validate SSA as a candidate neural correlate of the MMN. To study the topographic organization of SSA, we mapped the whole rat auditory cortex with MUA recordings from middle layers IIIb/IV using an oddball paradigm. We demonstrate that SSA occurs beyond A1, and its properties differ between primary and nonprimary fields. Our major findings are: (1) Highest SSA is sharply segregated to nonprimary fields, creating a distinct topographic gradient of SSA within the auditory cortex. (2) High SSA is present in nonprimary fields up to 200 ms after stimulus onset, and it remains stronger than in primary fields during the first 100 ms of the neuronal responses. (3) In all cortical fields, SSA is correlated in time and strength with the difference wave seen in both the fast (Nd) and slower (Pd) deflections of the LFP. As additional novel findings, we show that (4) SSA produces a delay in the responses to standard tones, as compared to deviants, and this delay is longer in nonprimary fields. (5) SSA is significantly higher for high frequencies of stimulation, and this dependence is more pronounced in primary fields. (6) SSA occurs faster and reaches a much lower plateau in the nonprimary fields.

**Fig 9:**
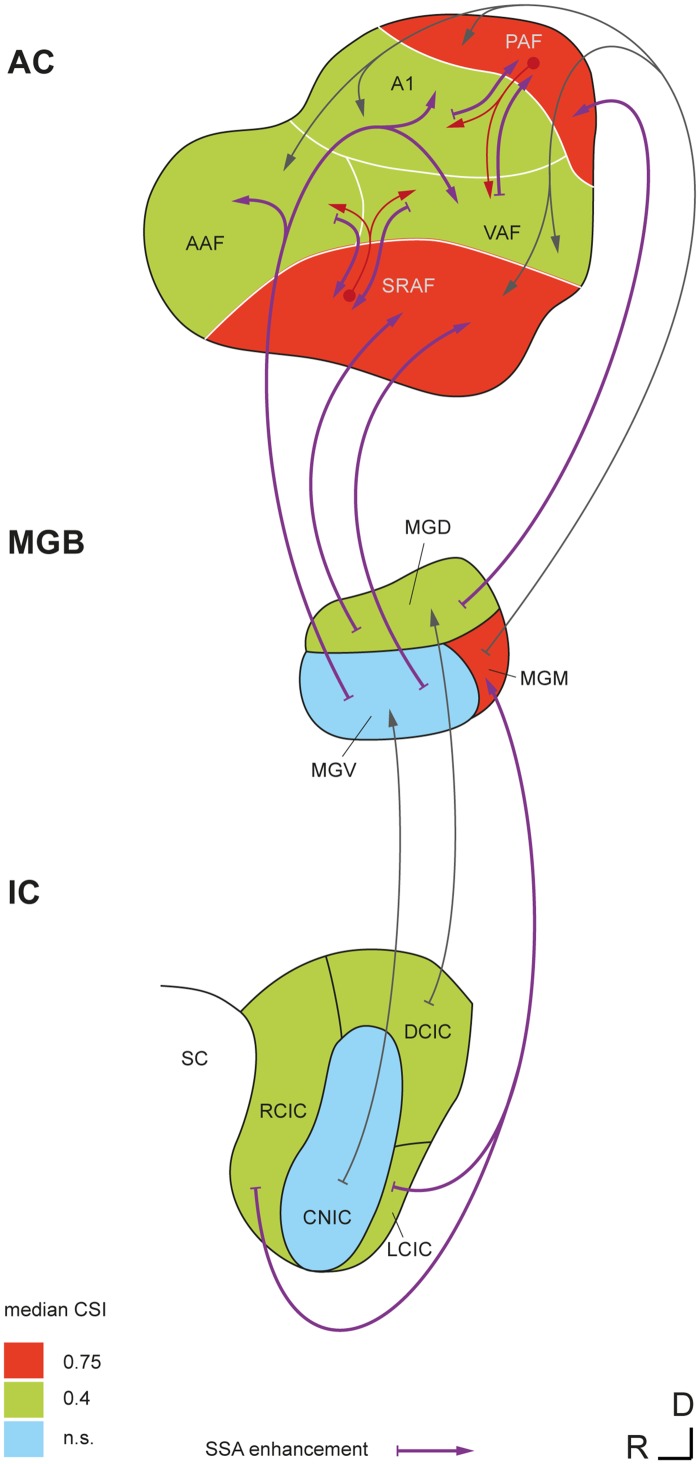
Emergence of SSA in the nonlemniscal auditory pathway. Simplified wiring diagram showing SSA levels and ascending connections of the auditory brain in which SSA occurs. SSA is virtually absent from lemniscal parts of the IC (central nucleus of the IC [CNIC]) and MGB (ventral division of the MGB [MGV]), but it is high in their nonlemniscal subdivisions (rostral, dorsal, and lateral cortices of the IC [RCIC, DCIC, and LCIC, respectively]; dorsal and medial divisions of the MGB [MGD and MGM, respectively]), showing levels comparable to those seen in primary cortical fields. Extreme levels of SSA are found only in nonprimary fields of the auditory cortex and in the MGM. Thus, SSA undergoes a significant enhancement at both lemniscal and nonlemniscal thalamocortical projections. A potential influence of nonprimary fields on high late-SSA seen in primary fields is represented by the red arrows. Median CSI values in the IC and MGB are from [17] and [15], using similar paradigm parameters.

One key aspect of our data is the high coincidence in the relative position of the fields across animals and in comparison with previous mapping studies [23,24,32,36]. Our analysis revealed a systematic meta-organization of SSA in the auditory cortex of the rat [23,36], such that the CSI gradient shows a steep increase at the boundaries between primary and nonprimary fields (Fig 4B). In particular, the sharp CSI enhancement between A1 and PAF (Fig 4D) bears striking resemblance with the same border found previously for bandwidth and latency [24]. Our results conform with previous studies that showed SSA in A1 [12,19,44–50] and extend their findings, as we present new SSA properties hitherto unknown. Importantly, the distribution of SSA indices in our A1 sample is largely equivalent to those shown in previous studies of SSA in the rat or mouse A1 that used similar paradigm parameters [19,47,50], making further comparisons more reliable. To the best of our knowledge, there were no previous studies of SSA outside A1, although higher SSA levels were expected to be found in nonprimary fields, since neurons in nonprimary cortical areas are known to show fast adaptation [20,21]. In particular, many studies independently reported that PAF neurons in the rat adapt strongly even to slow repetition rates [22–24], and novel sounds produced greater cellular activity than familiar sounds in auditory association cortex in area Te3 [51], where the SRAF is located [35]. There is also strong evidence of enhanced adaptation in nonprimary areas of the auditory cortex from large-scale brain responses (ERP, magnetoencephalography [MEG], fMRI) in both animals [28,29,52,53] and humans [25–27,54]. Our findings also parallel the topography of subcortical SSA (Fig 9). Previous studies consistently found stronger SSA in the nonprimary (or nonlemniscal) subdivisions of the IC [14–16] and MGB [17,55]. Importantly, an identical dependence of SSA on frequency of stimulation as well as a delay in onset latency of responses to standards have already been shown in the IC [15].

Our data sharply contrast with previous studies showing that the SSA level in A1 neurons is independent of their CF and in which less than 4% of neurons showed a latency effect [56]. However, the presence of strong SSA in spiking responses at 50–100 ms and beyond represents the major difference with previous SSA studies. Only very recently, two studies in mouse auditory cortex [49,50] and one in rat somatosensory cortex [57] found SSA in either subthreshold V_m_ fluctuations of layer II/III pyramidal neurons [49] or spiking responses of inhibitory interneurons [49,50] and layer IV pyramidal neurons [57] occurring more than 50–100 ms after stimulus onset. Importantly, we recorded mainly form layer IIIb/IV neurons, receiving direct thalamocortical inputs, which are more likely to show long-latency spiking responses [58]. Finally, previous studies reported SSA for LFP in A1, but they failed to show any correlation between MMN-like components of the LFP and SSA. Some did not find significant spiking activity for latencies beyond 50 ms [44,45] or observed SSA only for the fast Nd [46]; others did not measure MUA [59], or their analysis was restricted to the fast Nd only [19]. Such a correlation has only been described in the somatosensory cortex [57].

The mechanisms and location of the neural generators of SSA and their relation to MMN are still subjects of debate [11,13,60,61]. In the lemniscal pathway (Fig 9), SSA undergoes a first enhancement at the thalamocortical synapses from the ventral division of the MGB to A1 [12,17]. Here, we show a further enhancement of SSA in nonprimary cortical fields, which integrate the thalamocortical projection from nonlemniscal MGB [31] and the corticocortical projection from primary fields [62] and redirect their output to prefrontal and limbic brain regions involved in spatial attention and emotional memory [34,35]. Thus, our study confirms that SSA is a prevalent property of the nonlemniscal auditory pathway, even at the cortical level (Fig 9). This organization may underlie its functional significance as a higher-order stage of sensory processing beyond the faithful representation of the auditory stimuli that predominates in the lemniscal pathway [63]. Cumulating evidence indicates the existence of a hierarchy of processing stages for regularity encoding in the auditory brain, with later response components showing sensitivity for changes in more complex aspects of the acoustic scene [13,60,64]. Repetition positivity (RP) has been proposed as the electrophysiological correlate of the memory trace formation required for subsequent change detection and, in turn, rapid SSA in auditory cortex is likely to contribute to its generation [65,66]. Here, we show very strong SSA in nonprimary auditory cortex, supposed to contain the main generators of the MMN in humans [25,27,54,67,68], cats [53], and rats [29], that resembles MMN in several ways. First, SSA results in stronger responses to deviants than to standards in the oddball paradigm, to the extent that responses to standards can get totally suppressed in some recordings from nonprimary fields. Critically, we show strong SSA in these areas between 50 and 100 ms, correlated with a consistent difference wave at the slow Pd component of the LFP (Fig 8A). The latency of this Pd deflection (60–80 ms) is considerably shorter than the human MMN (150–200 ms) but matches perfectly the range of MMN-like potentials in the rat [28,29,69–73], which tend to occur, on average, 50–100 ms after stimulus onset, probably due to the smaller size of the rat brain [37]. Interestingly, this SSA resembles RP in the first standard presentations (Fig 7B) and matches stimulus statistics at multiple time scales [56,74]. We also show stronger SSA for high- than for low-frequency tones, paralleling a commonly observed effect of frequency in both animal [71–73] and human [75,76] MMN recordings. Therefore, we present strong evidence linking animal SSA to the human MMN, a result thus far missing in animal research. Importantly, we show that an MMN-like difference signal can readily result from SSA to standard tones that leaves responses to deviants unaffected (Fig 7A). Additionally, our LFP recordings show that the same components were present in responses to both standard and deviant tones (Fig 8A), consistent with the view that the MMN is a differentially adapted obligatory component of the ERPs. If so, our results would suggest a purely SSA explanation for the MMN [6,7,26].

Before we conclude, we should draw attention to three major caveats of our study. First, anesthesia reduces neuronal responsiveness to auditory stimuli as well as spontaneous firing, and may change some receptive field properties [77–79]; thus, an increased sensitivity to anesthetics in higher-order fields may lead to an overestimation of the SSA seen in those areas. However, we observed high spontaneous rates as well as strong, sustained responses to deviants in nonprimary fields (Fig 5A; baseline-corrected spike counts within 0 and 200 ms, mean ± SEM: A1, 3.2 ± 0.1; AAF, 2.8 ± 0.1; VAF, 4.7 ± 0.2; SRAF, 3.9 ± 0.2; PAF, 2.6 ± 0.2). We used urethane as anesthetic because it preserves balanced neural activity better than other agents [80], retains the higher-order processing capabilities of the auditory cortex [81], and shows no significant effects on SSA levels, at least in the IC [82]. Most importantly, MMN-like responses have been successfully recorded from anesthetized [29,69–71] and awake [28,72,73] animals alike (for review, see [10]). Second, the MMN is a negative-going component, in contrast to the positive late potential (Pd) examined here. Depending on the location of recording and anesthetic state, epidural MMN recordings in rats can be positive in polarity [72,73], an effect commonly observed in urethane-anesthetized preparations [69,71]. Moreover, an inversion of the LFP has been extensively described using laminar probes in A1 [45,59], such that positivities in layers IIIb/IV may appear as negativities in superficial layers. Third, there are some discrepancies between the SSA seen in MUA and in LFP data. Namely, whereas the MUA shows prominent activity between 100 and 200 ms (i.e., beyond the rat-MMN range), the LFP is relatively flat within this time window. Similar late-spiking activity has been observed in parvalbumin-positive inhibitory interneurons [49] and interpreted as delayed reverberating network activity specifically triggered by deviant stimuli, but we cannot rule out that MUA includes activity from thalamocortical afferents in layers IIIb/IV, which would not produce a prominent LFP component. Alternatively, the late enhancement of SSA (100–200 ms) seen in the primary fields (Fig 5B and 5C) might result from processing in the nonprimary fields, subsequently transmitted downwards through the massive feedback corticocortical connections (Fig 9) [34,35,57,83]. A more relevant discrepancy is that the difference-wave amplitude for the later Pd component of the LFP is comparable between primary and nonprimary auditory cortex and even significantly smaller in PAF than in A1 or AAF (Fig 8B), not supporting the notion of enhanced SSA in nonprimary fields. However, previous ERP studies [28,29] failed to find differences in the MMN amplitude between primary and nonprimary fields. One simple reason for this could be that ERPs and LFPs are large-scale potentials, reflecting overall synaptic activity within a wide volume of tissue [43], most probably spanning the boundaries between fields. Therefore, local measures at the cellular level, such as MUA, are much better indicators of specific differences between fields. Furthermore, it is consistent to find higher SSA at the MUA than at the LFP level (i.e., output versus input, respectively) within any particular area, as also shown at the single-neuron level [48]. Additionally, the amplitude of the difference wave is an absolute measure, whereas SSA is commonly expressed as a contrast, such as the CSI. When computed this way, SSA for the Pd amplitude is already higher in nonprimary than in primary fields, yet this difference is much sharper for the MUA, reflecting the operations carried out by nonprimary fields to their already-adapted inputs.

At this juncture, it is important to note that the slower Pd component of the difference wave peaked with the same latency throughout the entire auditory cortex (Fig 8B), and so did its epidural counterpart in the rat [29]. By contrast, the fast Nd deflection of the LFP occurs earlier in primary than in nonprimary fields (Fig 8B), suggesting a lemniscal origin and bottom-up propagation. Therefore, the higher degree of reciprocal interaction between fields is likely involved in the generation of the Pd, consistent with the idea that intracortical processing contributes to SSA at longer latencies [12,50,57,59,84]. Thus, MMN-like potentials may be readily recorded from both primary and nonprimary auditory cortex, but nonprimary fields seem to contribute critically to their generation at the microcircuit level [27,85].

In conclusion, we demonstrate that strong SSA occurs in nonprimary auditory cortex at the latency range of the MMN in the rat. This finding overcomes the two main discrepancies hitherto alleged against the suggestion that SSA in the auditory cortex may underlie the generation of the MMN [7,86], namely, its anatomical location and its temporal development. We provide empirical evidence of the missing link between SSA in single neurons and scalp-recorded potentials, thus bridging the gap between animal physiology studies and the human MMN. Given the wide use of the MMN as a tool in clinical and cognitive neuroscience [9,10,87,88], such a connection is potentially of high relevance for future research in these fields.

## Materials and Methods

### Surgical Procedures

The experimental protocols were approved by, and used methods conforming to the standards of, the University of Salamanca Animal Care Committee and the European Union (Directive 2010/63/EU) for the use of animals in neuroscience research. Experiments were performed on 12 adult female Long-Evans rats with body weights within 200 and 250 g. Surgical anesthesia was induced and maintained with urethane (1.5 g/kg, i.p.), with supplementary doses (0.5 g/kg, i.p.) given as needed. Dexamethasone (0.25 mg/kg) and atropine sulfate (0.1 mg/kg) were administered at the beginning of the surgery and every 10 h thereafter to reduce brain edema and the viscosity of bronchial secretions, respectively. Prior to surgery and recording sessions, we recorded auditory brainstem responses (ABR) with subcutaneous electrodes to ensure the animal had normal hearing. ABRs were collected using Tucker-Davis Technologies (TDT) software (BioSig) and hardware (RX6 Multifunction Processor) following standard procedures (0.1 ms clicks presented at a 21/s rate, delivered in 10 dB ascending steps from 10 to 90 dB SPL). The animal was then placed in a stereotaxic frame in which the ear bars were replaced by hollow specula that accommodated a sound delivery system.

After the animal reached a surgical plane of anesthesia, the trachea was cannulated for artificial ventilation and a cisternal drain was introduced to prevent brain hernia. Corneal and hind-paw withdrawal reflexes were monitored to ensure that a moderately deep anesthetic plane was maintained as uniformly as possible throughout the recording procedure. Isotonic glucosaline solution was administered periodically (5–10 ml every 6–8 h, s.c.) throughout the experiment to prevent dehydration. Body temperature was monitored with a rectal probe and maintained between 37 and 38°C with a homoeothermic blanket system (Cibertec). The skin and temporal muscles over the left side of the skull were reflected, and a 6 × 5 mm craniotomy was made in the left temporal bone to expose the entire auditory cortex. The dura was removed and the exposed cortex and surrounding area were covered with a thin, transparent layer of agar to prevent desiccation and to stabilize the recordings.

At the end of the surgery, a magnified picture (25×) of the exposed cortex was taken with a digital SLR camera (D5100, Nikon) coupled to the surgical microscope (Zeiss) through a lens adapter (TTI Medical). The picture included a pair of reference points previously marked on the dorsal ridge of the temporal bone, indicating the absolute scale and position of the image with respect to the bregma. This picture was displayed on a computer screen, and a micrometric grid was overlapped to guide and mark the placement of the electrode for every recording made. Recording sites (150–250 μm spacing; Fig 1A) were evenly distributed across the cortical region of interest while avoiding blood vessels. The vascular pattern was used as a local reference to mark the position of every recording site in the picture, but otherwise differed largely between animals. To confirm the actual depth and cortical layer of the recorded neurons, at the end of the experiment we made electrolytic lesions at one to four of the recording sites at the same depth that recordings were made.

### Electrophysiological Recording Procedures

Experiments were performed inside a sound-insulated and electrically shielded chamber. Sounds were generated using an RX6 Multifunction Processor (TDT) and delivered monaurally (to the right ear) in a closed system through a Beyer DT-770 earphone (0.1–45 kHz) fitted with a custom-made cone and coupled to a small tube (12-gauge hypodermic) sealed in the ear. The sound system response was flattened with a finite impulse response (FIR) filter, and the output of the system was calibrated in situ using a ¼-in condenser microphone (model 4136, Brüel & Kjær), a conditioning amplifier (Nexus, Brüel & Kjær), and a dynamic signal analyzer (Photon+, Brüel & Kjær). The output of the system had a flat spectrum at 76 dB SPL (±3 dB) between 500 Hz and 45 kHz, and the second and third harmonic components in the signal were ≤ 40 dB below the level of the fundamental at the highest output level (90 dB SPL) [14].

MUA was recorded with self-manufactured glass-coated tungsten electrodes (1–5 MΩ impedance at 1 kHz) [89,90]. A single electrode was positioned orthogonal to the pial surface (forming a 30° angle with the horizontal plane) and advanced 350–550 μm into the thalamorecipient layers IIIb–IV using a piezoelectric micromanipulator (Sensapex) until we observed a strong spiking activity synchronized with the train of searching stimuli. The signal was amplified (1000×) and band-pass filtered (1 Hz to 3 kHz) with a differential amplifier (DAM-80, WPI). This analog signal was digitized at a 12K sampling rate and further amplified and band-pass filtered for action potentials (between 500 Hz and 3 kHz). Spike waveforms and relative times in respect to the start of the recording were displayed and stored in a PC running Windows XP (Microsoft). A bilateral threshold for automatic action potential detection was set at about two to three standard deviations of the background noise. In a subset of the experiments, the digital signal was further filtered for LFP (between 3 and 50 Hz), decimated to a 508 Hz sampling rate and stored in continuous form for posterior analysis. Stimulus generation and neuronal response visualization were controlled online with custom software created with the OpenEx suite (TDT) and Matlab (Mathworks).

Sounds used for stimulation were white noise bursts or pure tones with 5 ms rise-fall ramps. Sounds used for searching for neuronal activity were trains of noise bursts or pure tones (1–8 stimulus per second). We used short stimulus duration for searching (30 ms) to prevent strong adaptation. In addition, type (noise, pure tone) and parameters (frequency, intensity, presentation rate) of the search stimuli were varied manually when necessary to facilitate release from adaptation and, thus, prevent overlooking responses with high SSA. Once a suitable recording site was reached, the FRA was determined using 75 ms pure tones at varying frequencies and intensities (Fig 2A; 0.5–44 kHz logarithmically spaced at 0.25 octave steps, 0–70 dB SPL at 10 dB steps, 375 ms onset-to-onset interval, one to three randomized repetitions of each stimulus). The FRA was displayed on a computer screen using custom software, and the frequency-tuning curve was automatically outlined as the minimum sound intensity that elicited a firing rate over 20%–40% of the maximum firing for each frequency. Thus, the minimum response threshold and CF were computed for each site (excluding isolated “islands” of spontaneous activity), and two frequencies (*f*_*1*_, *f*_*2*_) were selected to use in the oddball paradigm [12] at 20–30 dB above threshold. The two stimuli were selected so as to evoke strong responses of similar magnitude at that recording site. In some cases, one or more extra pairs of stimuli were selected to ensure at least one recording met this requirement. Two oddball sequences with fixed parameters (250 trials each, 75 ms stimulus duration, 0.5 octaves frequency separation, 10% deviant probability, 300 ms onset-to-onset interval, minimum of three standard tones before a deviant) were presented for every pair of stimuli thus selected. In one of the sequences, the low frequency (*f*_*1*_) was the “standard” and the high frequency (*f*_*2*_) was the “deviant,” and in the other sequence their roles were swapped. The order of presentation of these two sequences was randomized across sites.

### Data Analysis

Peristimulus time histograms (PSTH) were generated for every stimulus and condition tested. Only the last standard tones preceding each deviant were used for the analyses, except for the time course analysis, where all standard trials were analyzed. Every PSTH was analyzed to test for significant auditory responses and to extract several different metrics of response strength and latency. For these analyses, the original PSTH was smoothed with a 6 ms gaussian kernel (“ksdensity” function in Matlab) in 1 ms steps to estimate the spike-density function (SDF) over time, and the baseline spontaneous firing rate (SFR) was determined as the average firing rate during the 75 ms preceding stimulus onset. For any given time window, the excitatory response was measured as the area below the SDF and above the baseline SFR. This measure will be referred to as “baseline-corrected spike count” (BCSC). To test for statistical significance of the BCSC we used a Monte Carlo approach. First, 1000 simulated PSTHs were generated using a Poisson model with a constant firing rate equal to the SFR. Then, a “null distribution” of BCSC was generated from this collection of PSTHs, following these same steps. Finally, the *p*-value of the original BCSC was empirically computed as *p* = (*g* + 1) / (*N* + 1), where *g* is the count of “null” measures greater than or equal to BCSC and *N* = 1000 is the size of the “null” sample. Note that using this approach, the minimum *p*-value that can be obtained is 1/1001 ≈ 0.001.

When a significant evoked activity was detected, onset and offset latencies of the whole excitatory response were computed as follows. First, a “noise” threshold was computed, as the firing rate below which the pure-spontaneous simulated SDFs remained 97.5% of the time. Every SDF, including the simulated ones, was scanned for stretches of “signal” above this threshold, and the amount of “signal” for each stretch was measured as the area below the SDF and above the SFR during that particular interval. Using the distribution of all the signal stretches thus found within the 1,000 pure-spontaneous SDFs, a Monte Carlo test was used to compute empirical *p*-values for every stretch of signal found in the target SDF under study. For each significant signal stretch (*p* < 0.05), the start/end times (*T*_*on*_, *T*_*off*_) were determined as the time points when the SDF trace cuts the noise threshold, and onset/offset latencies of the whole excitatory response (*ONSET*, *OFFSET*) were defined as the *T*_*on*_/*T*_*off*_ of the first/last significant excitatory component of the response, respectively. Peak firing rate amplitude was defined as the maximum firing rate reached by the SDF within the analysis window, minus the SFR baseline, and peak latency as the time point respect stimulus onset that this peak takes place. Finally, the duration of the whole significant response interval was defined as *OFFSET*–*ONSET*, and the duration of the strong peak of the response, or “half-peak response duration,” was measured as the total length of time that the SDF remains above 50% of the peak amplitude.

In order to quantify and compare SSA levels between the five fields, we computed the frequency-specific SSA index for each stimulus, SI(*f*_*1*_) and SI(*f*_*2*_), and the common SSA index (CSI) for every recording site in the usual way [12]:

$$SI\left(f_{i}\right)=\frac{DEV\left(f_{i}\right)-STD\left(f_{i}\right)}{DEV\left(f_{i}\right)+STD\left(f_{i}\right)}\;;\;i=1,2$$

$$CSI=\frac{\sum DEV\left(f_{i}\right)-\sum STD\left(f_{i}\right)}{\sum DEV\left(f_{i}\right)+\sum STD\left(f_{i}\right)}\;;\;i=1,2$$

Where DEV(*f*_*i*_), STD(*f*_*i*_) are baseline-corrected spike counts in response to frequency *f*_*i*_ when it was a deviant and standard, respectively. The CSI was calculated only for recordings with significant auditory responses to at least one frequency in the oddball paradigm (either as deviant or as standard). In cases in which more than one stimulus pair was tested at the same recording site, we selected only one to compute SSA for that site, according to the following criteria: (1) Recordings with significant responses to both frequencies (either as deviant or as standard) were always preferred to recordings with significant response to only one of them. (2) We selected the recording with most similar responses to *f*_*1*_ and *f*_*2*_ (as deviants); the similarity between responses was measured as their ratio, *f*_*1*_/*f*_*2*_ or *f*_*2*_/*f*_*1*_, whichever was less than 1. (3) If there were two or more recordings with similar deviant-to-deviant ratios (difference of ratios < 0.1), we selected the one with the lowest sound level (SPL) used for stimulation.

For the analysis of the LFP signal, we aligned the recorded wave to the onset of the stimulus for every trial and computed the mean LFP for every recording site and stimulus condition (deviant, standard) as well as the difference wave (DW = deviant−standard). Then, grand-averages were computed for deviant, standard, and DW across the whole auditory cortex and for every field separately. The *p*-value of the grand-averaged DW was determined for every time point with a two-tailed *t* test, Bonferroni-corrected for 204 comparisons (overall significance level of 0.05), and the time intervals in which a significant DW was observed were computed. For each individual (mean) LFP wave, the peak amplitude and latency were computed within two time windows: [10–40 ms] and [50–90 ms], corresponding to the first Nd and second Pd seen in the grand-averages within all fields. When comparing response features between fields, such as onset latency or CSI, we used nonparametric Kruskall-Wallis or Friedman tests, given the non-normal nature of these measures. Each of these tests was followed by a post-hoc multiple comparison test, using the Dunn-Sidak method at a 5% significance level, to detect specific differences between fields. For the sake of readability, *p*-values for all tests are reported using an upper bound equal to the minimum power of ten or half a power of ten that is greater than the actual *p*-value (e.g., *p* < 5·10^−6^).

For the time course analysis, we first computed the average standard and deviant response at each absolute position within the sequence for all neurons tested within each cortical field separately. A single-trial spike count for any given PSTH was computed as the number of spikes between the previously determined *ONSET* and *OFFSET* times, minus the baseline SFR. Then, we fitted these time series to different models (linear, exponential, double exponential, polynomial inverse, and power law with two or three coefficients) using the “fit” function in Matlab, which also computes the coefficient of determination (adjusted-r^2^) of the whole fit and confidence intervals for the fitted parameters.

To quantify the topographical organization of a feature map and test for statistical significance thereof, we used the “MapTools” library in Matlab, applying the topographic product statistic [39]. This metric was used instead of other alternatives (Pearson and Spearman linear correlation, Zrehen measure, etc.) due to the highly non-normal nature of the data under study (i.e., CSI) and assuming a local, linear nature of the topography of the CSI. To generate averaged maps for CF, CSI, and other response features, we followed a spotlight-average approach: starting with the set of sample points in which actual recordings were made and the associated values of the feature, we computed the averaged feature value for any other point in the map from its nearest neighbors. Specifically, we placed a bivariate Gaussian kernel of 100-μm radius,

$$\text{ker}\left(x,y\right)=\frac{1}{2\pi r}\cdot \text{exp}\left\{\frac{\sqrt{x^{2}+y^{2}}}{2r^{2}}\right\},$$

centered on every sample point and multiplied it by its associated feature value. Then we summed all these functions over the entire map and divided the result by the sum of all kernels at every point, to compute a weighted average throughout the whole surface. Thus, the feature value *V* at every point of the map was calculated as:

$$V(x,y)=\frac{\Sigma_{i=0}^{n}v_{i}\cdot ker(x-x_{i},y-y_{i})}{\Sigma_{i=0}^{n}\;ker(x-x_{i},y-y_{i})},$$

where *x*, *y* are the coordinates of a generic point in the map, and *x*_*i*_, *y*_*i*_ (*i* = 1, …, n) are the sample points used to generate the map. To impose a limit on the influence span for every point, this weighted average was computed only for points where the sum of all kernels (denominator in the last formula) was greater than 0.05. Further, to avoid single-point averages, we computed *V(x*,*y)* only when at least two neighboring sample points had been used for averaging.

To combine data from different animals, we followed an iterative process to improve the quality of the alignment in successive stages. We first generated the CF map for the case with the greatest number of recordings (shown in Fig 1). Then, we applied a manual shift to each of the remaining maps in turn so as to put them into register with the former. We used the CF gradient, the “unresponsive spot” at the wedge between A1, AAF, and VAF, and the low-frequency centers in A1, AAF, and SRAF as main references to determine, for each animal, the absolute position of the map with respect to the bregma [23]. Finally, we computed the topographic product statistic for the whole set of aligned recordings. This alignment was refined and the test statistic was recalculated until no improvement was detected in the correlation. We repeated this process for every animal until the alignment was completed.

## Supporting Information

S1 Data Full dataset used in reported results and figures. Organized into four spreadsheets: “Recording site,” list of all MUA recordings made, including those that didn’t show good responses to pure tones or otherwise could not be tested with the oddball paradigm; “Oddball (MUA),” list of all MUA recordings that could be tested with the oddball paradigm (one selected recording per multiunit), contains main stimulation parameters, response measures, and SSA indexes; “Time Course,” contains trial-by-trial spike counts used to fit the time course of SSA and generate Fig 7; “Oddball (LFP),” list of all recording sites with LFP recordings made, contains main responses and adaptation measures reported for the Nd and Pd deflections of the LFP.
(XLSX)

S2 Data Selected dataset (full SSA index sample) underlying Fig 3. (XLSX)

S3 Data Selected dataset used to generate the maps in Fig 4. Contains separate spreadsheets for Fig 4A (map of CF, all recordings with a well-defined CF) and Fig 4B–4D (maps of adaptation, all recordings tested with the oddball paradigm).
(XLSX)

S4 Data Selected dataset (CSI measures at different time windows) underlying boxplots and maps in Fig 5B and 5C. (XLSX)

S5 Data Selected dataset (tone frequency, intensity, firing rate, and SI for each tone [*f1* and *f2*, separately]) used to fit the linear model for the SI described in Results and to generate SI maps in Fig 6. (XLSX)

S6 Data Average trial-by-trial spike counts used to fit the time course of SSA within each field and generate Fig 7. (XLSX)

S7 Data Selected dataset (amplitude and latency of the Nd and Pd difference) underlying barplots in Fig 8B. (XLSX)

S8 Data Selected dataset (SSA for the Nd and Pd components) reported in Results but not shown in any figure. (XLSX)

## Abbreviations

AAF: anterior auditory field
A1: primary auditory cortex
CF: characteristic frequency
CNIC: central nucleus of the inferior colliculus
CSI: common stimulus-specific adaptation index
DCIC: dorsal cortex of the inferior colliculus
DW: difference wave
ERP: event-related potential
FRA: frequency response area
IC: inferior colliculus
LCIC: lateral cortex of the inferior colliculus
LFP: local field potential
MEG: magnetoencephalography
MGB: medial geniculate body
MGD: dorsal division of the MGB
MGM: medial division of the MGB
MGV: ventral division of the MGB
MMN: mismatch negativity
MUA: multiunit activity
Nd: negative deflection of the LFP
PAF: posterior auditory field
Pd: positive deflection of the LFP
RCIC: rostral cortex of the inferior colliculus
RP: repetition positivity
SI: stimulus-specific adaptation index
SRAF: suprarhinal auditory field
SSA: stimulus-specific adaptation
VAF: ventral auditory field
